# Involvement of Fenton chemistry in rice straw degradation by the lignocellulolytic bacterium *Pantoea ananatis* Sd-1

**DOI:** 10.1186/s13068-016-0623-x

**Published:** 2016-10-06

**Authors:** Jiangshan Ma, Keke Zhang, Mei Huang, Stanton B. Hector, Bin Liu, Chunyi Tong, Qian Liu, Jiarui Zeng, Yan Gao, Ting Xu, Ying Liu, Xuanming Liu, Yonghua Zhu

**Affiliations:** 1 Hunan Province Key Laboratory of Plant Functional Genomics and Developmental Regulation, College of Biology, Hunan University, Changsha, 410008 Hunan People’s Republic of China; 2 Department of Genetics, Institute for Plant Biotechnology, Stellenbosch University, Private Bag X1, Matieland, 7602 South Africa; 3 DNA Sequencing Unit, Central Analytical Facility, Stellenbosch University, Private Bag X1, Matieland, 7602 South Africa

**Keywords:** Lignocellulose degradation, *Pantoea ananatis* Sd-1, Fenton chemistry, Rice straw, Bacteria degradation system

## Abstract

**Background:**

Lignocellulolytic bacteria have revealed to be a promising source for biofuel production, yet the underlying mechanisms are still worth exploring. Our previous study inferred that the highly efficient lignocellulose degradation by bacterium *Pantoea ananatis* Sd-1 might involve Fenton chemistry (Fe^2+^ + H_2_O_2_ + H^+^ → Fe^3+^ + OH· + H_2_O), similar to that of white-rot and brown-rot fungi. The aim of this work is to investigate the existence of this Fenton-based oxidation mechanism in the rice straw degradation process of *P. ananatis* Sd-1.

**Results:**

After 3 days incubation of unpretreated rice straw with *P. ananatis* Sd-1, the percentage in weight reduction of rice straw as well as its cellulose, hemicellulose, and lignin components reached 46.7, 43.1, 42.9, and 37.9 %, respectively. The addition of different hydroxyl radical scavengers resulted in a significant decline (*P* < 0.001) in rice straw degradation. Pyrolysis gas chromatography–mass spectrometry and Fourier transform infrared spectroscopy analysis revealed the consistency of chemical changes of rice straw components that exists between *P. ananatis* Sd-1 and Fenton reagent treatment. In addition to the increased total iron ion concentration throughout the rice straw decomposition process, the Fe^3+^-reducing capacity of *P. ananatis* Sd-1 was induced by rice straw and predominantly contributed by aromatic compounds metabolites. The transcript levels of the glucose-methanol-choline oxidoreductase gene related to hydrogen peroxide production were significantly up-regulated (at least *P* < 0.01) in rice straw cultures. Higher activities of GMC oxidoreductase and less hydrogen peroxide concentration in rice straw cultures relative to glucose cultures may be responsible for increasing rice straw degradation, which includes Fenton-like reactions.

**Conclusions:**

Our results confirmed the Fenton chemistry-assisted degradation model in *P. ananatis* Sd-1. We are among the first to show that a Fenton-based oxidation mechanism exists in a bacteria degradation system, which provides a new perspective for how natural plant biomass is decomposed by bacteria. This degradative system may offer an alternative approach to the fungi system for lignocellulosic biofuels production.

**Electronic supplementary material:**

The online version of this article (doi:10.1186/s13068-016-0623-x) contains supplementary material, which is available to authorized users.

## Background

Plant biomass, the most abundant terrestrial carbon source, represents a valuable feedstock for lignocellulosic biofuels [1]. To achieve complete transformation of plant biomass, a key issue is to efficiently disrupt the recalcitrant barriers of plant lignocellulose structure thus releasing fermentable sugars as well as other valuable chemicals. A number of microorganisms can carry out biomass conversion via secretion of polysaccharide- and/or lignin-degrading enzymes [2, 3]. However, these enzymes are usually too large to penetrate intact plant cell walls, leading to chemical or physical pretreatment of biomass prior to microbial conversion [4, 5]. Nevertheless, a few of other microorganisms were found to be able to decompose natural lignocellulosic biomass without pretreatment. Comparing to the high energy input, harsh conditions, and toxic residues of most chemical and/or physical pretreatment strategies, these microbial conversions can be conducted under milder conditions and are more environmentally friendly. The white-rot fungi (notably *Phanerochaete chrysosporium*) and the brown-rot fungi (such as *Serpula lacrymans*) are classified under these kinds of microorganisms. Both these organisms have been extensively studied for their lignocellulose biodegradation mechanisms [2, 6–9]. It is inferred that white-rot and brown-rot fungi contain a Fenton-based oxidation model to modify the lignocellulose backbone to make it amenable to enzymatic hydrolysis [10, 11]. Hydroxyl radical (OH·), generated extracellularly via the Fenton chemistry (Fe^2+^ + H_2_O_2_ + H^+^ → Fe^3+^ + OH· + H_2_O), is such an oxidation agent [9, 12]. These hydroxyl radicals randomly attack lignocellulose at close proximity and disrupt its structural matrix through internal cleavage of cellulose chains and modification of lignin [13]. Besides, some reports have shown the existence of this Fenton-based oxidation mechanism in other fungi. For example, Xie et al. [14] showed that the Fenton system-mediated hydroxyl radicals play an important role in the lignin degradation process of oleaginous fungus *Cunninghamella echinulata* FR3. In recent years, the emerging role of bacteria in lignocellulose degradation has been the focus of much research attention, yet its mechanism is less well-studied compared to that of lignocellulolytic fungi. Previous studies have shown molecular weight similarities between bacterial and fungal lignocellulolytic enzymes [15], hence it is expected that bacteria may possess analogous Fenton-like oxidation mechanisms. However, to date, no reports have documented Fenton-based oxidation in bacteria lignocellulose conversion system.

In our previous study, we isolated an endophytic bacterium *Pantoea ananatis* Sd-1 (China General Microbiological Culture Collection Centre, no. 6698) and found that it has the capacity to simultaneously degrade cellulose, hemicellulose, and lignin [16]. To our knowledge, these characteristics have only been reported for very few microorganisms especially white-rot fungi. We demonstrated that *P. ananatis* Sd-1 was capable of secreting a plethora of lignocellulolytic enzymes while growing on rice straw-containing media. In addition, bioinformatic analysis inferred that a Fenton-like reaction mechanism might exist as a part of the *P. ananatis* Sd-1 lignocellulose degradation system [17]. In this study, in order to confirm Fenton chemistry-assisted degradation in *P. ananatis* Sd-1, we evaluated the activity of *P. ananatis* Sd-1 on natural rice straw as well as in the presence of different hydroxyl radical scavengers, including dimethylsulfoxide (DMSO), mannitol, thiourea, and ethanol. We analyzed chemical structure variations of rice straw after treatment with *P. ananatis* Sd-1 by pyrolysis gas chromatography–mass spectrometry (Py-GC/MS) and Fourier transform infrared (FTIR) spectroscopy. In addition, we assessed the total iron ion concentration in the system and Fe^3+^-reducing capacity of *P. ananatis* Sd-1, and verified the presence of low molecular weight products such as Fe^3+^-reductant by gas chromatography–mass spectrometry (GC–MS) analysis. Furthermore, gene and enzymes expression levels responsible for hydrogen peroxide generation and hydrogen peroxide concentration were also evaluated.

## Results and discussion

### Natural rice straw degradation characteristics of *P. ananatis* Sd-1

**Fig. 1:**
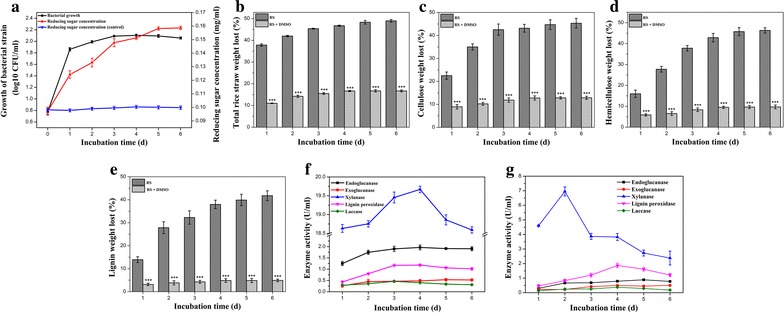
Growth of bacterial strain and reducing sugars production (**a**); percentage of weight loss for total rice straw (**b**), cellulose (**c**), hemicellulose (**d**), and lignin (**e**) in the presence (RS + DMSO) and absence (RS) of DMSO. Enzymes activities of endoglucanase, exoglucanase, xylanase, lignin peroxidase, and laccase in the absence (**f**) and presence of DMSO (**g**) during the degradation of rice straw by *P. ananatis* Sd-1. Control: non-inoculated cultures. The values represent the means of three replicates with the standard deviation (SD). The *asterisks* attached to data points represent statistically significant differences when DMSO was added versus not added. (statistical significance: ***P < 0.001)

Our previous report on the lignocellulose degradation capacity of *P. ananatis* Sd-1 was based on alkaline-pretreated rice straw [16]. In order to determine whether *P. ananatis* Sd-1 was able to degrade the natural lignocellulosic substrates, the bacterium was cultured on unpretreated [except underwent necessary autoclaving, because sterilization did not liberate more reducing sugar from rice straw to liquid than that of the unsterilized medium (data not shown)] rice straw medium and the resulting reducing sugars were measured. As shown in Fig. 1a, *P. ananatis* Sd-1 grew optimally in rice straw medium during the first 3 days of incubation and correspondingly, large yields of the reducing sugars were observed compared with that of non-inoculated control. In addition, the mass of the total rice straw (Fig. 1b), cellulose (Fig. 1c), hemicellulose (Fig. 1d), and lignin (Fig. 1e) contents all decreased substantially in the initial 3 days of treatment with the percentage weight loss reaching 46.7, 43.1, 42.9, and 37.9 %, respectively, while they changed almost nothing in control (data not shown). Interestingly, this is directly proportionate to the bacterial growth and the reducing sugar production. After the initial period, reducing sugar content increased slightly while there was a decline in bacterial growth. Equally, the weight reductions of total rice straw, cellulose, hemicellulose, and lignin were only 2.2, 2.2, 3.4, and 3.6 % respectively between 3 and 6 days. These results indicate that *P. ananatis* Sd-1 is able to partially depolymerize natural rice straw into its reducing sugars components. To date, very few bacteria have been reported to possess the capacity to decompose natural lignocellulosic substrates [2]. Kumar et al. [18] demonstrated that the maximum weight reduction of 49 % was observed for *Pseudoxanthomonas* sp. R-28 on day 3 when incubated with unpretreated rice straw. Wang et al. [19] reported that the unpretreated rice straw was degraded most expeditiously by bacterial consortium XDC-2 before day 6 with the weight loss of rice straw and its cellulose, hemicellulose, and lignin components reaching 39.6, 9.7, 84.4, and 30.2 %, respectively. Our evidences suggest that *P. ananatis* Sd-1 possesses comparable natural rice straw bioconversion abilities to these reported bacteria.

Our previous research showed *P. ananatis* Sd-1 to possess an arsenal of enzymes required for cellulose, hemicellulose, and lignin degradation [17]. Activities of several of these lignocellulolytic enzymes (endoglucanase, exoglucanase, xylanase, lignin peroxidase, and laccase) were measured during the rice straw degradation process. As shown in Fig. 1f, in correlation with the rapid degradation of rice straw, increased activities were observed for all detected enzymes during the logarithmic phase, i.e., the first 3 days of incubation. However, maximum activity of most of enzymes was reached on day 4 for endoglucanase, xylanase, and lignin peroxidase enzymes with the exception of the exoglucanase which reached optimal activity on day 5. Analysis of these results suggested that in addition to enzymatic hydrolysis, another oxidation pathway at the incipient stage of rice straw degradation in *P. ananatis* Sd-1 may exist. It is assumed that during this process, the rice straw structure weakens and exposes its cellulose, hemicellulose, and lignin components to relevant lignocellulolytic enzymes, giving rise to induction and increased expression of these enzymes.

With regards to laccase enzymes, it was speculated that it may oxidize some low molecular weight mediators to diffusible free radicals which could act as direct oxidants of lignocellulose in vitro; however, these mediators were not detected [20]. In our study, the maximum laccase activity was reached on day 3, which is in correlation with this proposed oxidization role for laccases. However, the detected laccase activity was maintained at much lower levels (maximum value 0.46 U/ml) compared to that of other reported bacteria (normally about 1 U/ml) [21–23]. This indicated that laccase might play a partial role in the oxidation of lignocellulose. These results support our hypothesis that another oxidation mechanism might assist *P. ananatis* Sd-1 degradation of rice straw.

### Influence of hydroxyl radical scavenger on rice straw degradation efficiency of *P. ananatis* Sd-1

Fenton-based oxidation via hydroxyl radicals generated by Fenton chemistry has been found in fungi for plant cell wall decomposition [12]. Jung et al. [24] also applied Fenton chemistry using FeCl_3_ and hydroxyl peroxide to pretreat rice straw. In order to verify whether a Fenton-like reaction exists as part of the process of rice straw degradation by *P. ananatis* Sd-1, we tested if the degradative efficiency is hydroxyl radical dependent. DMSO has been widely used as a hydroxyl radical scavenger [25]. The weight loss of rice straw (Fig. 1b) and its components (Fig. 1c–e) was significantly (*P* < 0.001) decreased in the presence of DMSO relative to that in the absence of DMSO, indicating the involvement of hydroxyl radicals in lignocellulose degradation. Bacterial growth remained unaffected, while reducing sugar production decreased due to the presence of DMSO (Additional file 1: Figure S1). Compared to the result in the absence of DMSO (Fig. 1a), we ruled out the possibility that the observed inhibition of DMSO on lignocellulose degradation was due to its toxicity on bacterium. Similar effects of DMSO were demonstrated by Grinhut et al. [13] during humic acid bleaching by *Trametes* sp. M23 which involved Fenton-like reaction mechanisms. Forney et al. [26] also reported that lignin degradation by *P. chrysosporium* which generated hydroxyl radicals via Fenton chemistry was markedly suppressed in the presence of the hydroxyl radical scavengers, benzoate, and mannitol. We also detected the effects of several other hydroxyl radical scavengers including mannitol, thiourea, and ethanol on rice straw degradative efficiency of *P. ananatis* Sd-1. Similar to that of DMSO, the degradation efficiencies of rice straw as well as its components were all significantly suppressed by these three hydroxyl radical scavengers after 6 days of incubation (Additional file 2: Figure S2a). These results added certainty that our conclusions were not an artifact of some other aspect of DMSO-related chemistry.

Fenton reaction can also affect the sugar production and degradation during the lignocellulose degradation [27]. Sugars (glucose, xylose, and arabinose), furfural (derived from pentoses), and 5-hydroxymethylfurfural (HMF, derived from hexoses) during the *P. ananatis* Sd-1 and Fenton reagent treatment are shown in Additional file 3: Table S1. Sugars and furfural concentrations all decreased in the presence of DMSO relative to that in the absence of DMSO during the incubation time. The result from Fenton reagent treatment is similar to that of the first two days of *P. ananatis* Sd-1 treatment. It is worth noting that, when DMSO was added, the appearance of methanesulfinic acid (product from the reaction of DMSO and hydroxyl radical) in the first 3 days of incubation was observed and the maximum of it was detected on day 2 (Additional file 4: Figure S3). These results provide evidences for the suggestion mentioned above that Fenton reactions play a pivotal role at the incipient stage of the plant cell wall decomposition as part of the degradation repertoire of *P. ananatis* Sd-1.

The effect of DMSO on lignin degradation (Fig. 1e) (an average of 85.6 % decrease) is higher than that of cellulose (Fig. 1c) and hemicellulose (Fig. 1d) (an average of 69.4 and 75.6 % decrease, respectively), highlighting the involvement of hydroxyl radicals in lignin modification. This was in contrast to reports that Fenton chemistry contributed more in cellulose degradation than in modifying and degrading lignin in several white-rot fungi with cellobiose dehydrogenase-mediated Fenton chemistry [28, 29]. The role of cellobiose dehydrogenase includes activating hydrolytic cellulases and iron reduction for Fenton chemistry in those white-rot fungi [30], while we found no genes encoding cellobiose dehydrogenase in the genome of *P. ananatis* Sd-1 [17]. Thus we speculated that the reason of the different effects of Fenton chemistry contribution on cellulose and lignin degradation between *P. ananatis* Sd-1 and those white-rot fungi might be the diverse iron reduction system between them.

It should be noted that DMSO was reported to affect the activities of lignocellulolytic enzymes [31]. As shown in Fig. 1g, the activities of endoglucanase, exoglucanase, xylanase, and laccase decreased average of 61.5, 14.6, 78.6, and 31.1 %, respectively, while lignin peroxidase activity increased average of 27.2 %, in presence of DMSO during the degradation process of rice straw by *P. ananatis* Sd-1. Besides, those enzymes activities were all suppressed at various extents in the presence of other three hydroxyl radical scavengers (mannitol, thiourea, and ethanol) (Additional file 2: Figure S2b–d). This suggested that in addition to the reduction of hydroxyl radical, the hydroxyl radical scavengers might also affect the lignocellulolytic enzymes activities and thus inhibit the rice straw degradation by *P. ananatis* Sd-1.

### Chemical structure variation of lignocellulose in rice straw after *P. ananatis* Sd-1 treatment

**Fig. 2:**
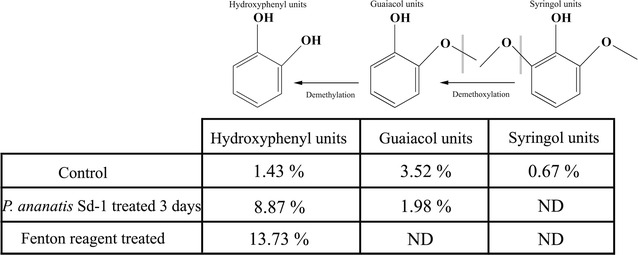
Py-GC/MS analysis of rice straw. Relative peak areas of lignin-subunit pyrolysates detected by Py-GC/MS from rice straw without treatment (control), after Fenton reagent treatment and 3 days of *P. ananatis* Sd-1 treatment. *ND* means not detected

Reaction to hydroxyl radicals can result in several alterations to lignin units, including side chain oxidation, hydroxylation, demethylation, and demethoxylation [9]. In order to obtain detailed insight into the chemical changes in the lignin structure of rice straw treated with *P. ananatis* Sd-1, Py-GC/MS analysis was performed. Pyrolysis products from rice straw lignin are classified as hydroxyphenyl, guaiacol, and syringol units according to their phenyl group structure. As shown in Fig. 2, Syringol was undetected in residual rice straw after *P. ananatis* Sd-1 or Fenton reagent treatment. The content of hydroxyphenyl relative to guaiacol increased after *P. ananatis* Sd-1 treatment (4.47 vs 0.41 of control). Fenton reagent caused guaiacol to disappear while the hydroxyphenyl occupied a much higher concentration compared to the control. These results indicated that both lignin modification by *P. ananatis* Sd-1 as well as Fenton reagent were mainly through demethylation and demethoxylation reactions. Similarly, Filley et al. [32] reported Fenton chemistry demethylation of lignin in spruce sapwood when degraded by brown-rot fungi, *Postia placenta* and *Gloeophyllum trabeum*. Rineau et al. [33] found that syringyl units in the organic matter from forest litter were both demethoxylated and demethylated by *Paxillus involutus* through Fenton chemistry. However, as aforementioned that laccase in *P. ananatis* Sd-1 might also play a partial role in the oxidation of lignocellulose including lignin demethylation, further studies are needed to distinguish whether the demethylation of lignin is caused by Fenton chemistry or laccase.

In addition, the Fourier transform infrared (FTIR) spectra of both Fenton reagent and *P. ananatis* Sd-1 treated rice straw gave similar characteristic transmission bands (Additional file 5: Figure S4). Compared to the control, the appearance of an additional peak (~1730 cm^−1^) (as black arrow indicated) located in the carbonyl groups region was also indicative of oxidative degradation through Fenton-like reaction. Since the formation of carbonyl groups can be attributed to ring opening reactions of polysaccharides and aromatic moieties within lignin by oxidation reaction [34], this is similar to the previous report of Rineau et al. [33], which detected sharp carbonyl groups signal in the FTIR spectrum of *Paxillus involutus* treated cellulose which was shown to be associated with the Fenton reaction mechanism. Consequently, cumulative findings allude to the existence of Fenton chemistry system in *P. ananatis* Sd-1.

### Induced Fe^3+^-reducing activity in *P. ananatis* Sd-1 degradation system

**Fig. 3:**
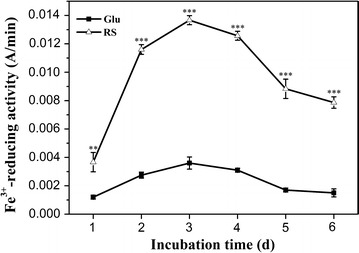
Time course of Fe^3+^-reducing activity of *P. ananatis* Sd-1 grown on glucose and rice straw substrates. *Glu* glucose substrate, *RS* rice straw substrate. The values represent the means of the three replicates with the standard deviation (SD). The *asterisks* attached to data points represent statistically significant differences from the glucose medium (statistical significance: **P < 0.01, ***P < 0.001)

**Fig. 4:**
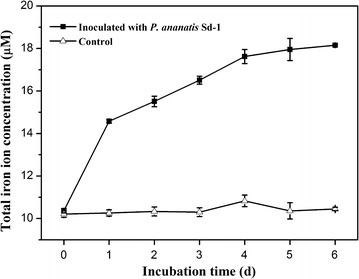
The variation of total iron ion concentration in cultures during the degradation process of rice straw with *P. ananatis* Sd-1 and without inoculation (control)

Fenton chemistry involves a redox cycling process, and a crucial factor is a system for reduction of Fe^3+^ to Fe^2+^ [35]. Fe^2+^ then reacts with hydrogen peroxide to form hydroxyl radical [36]. Thus, the hydroxyl radical level is influenced by Fe^3+^-reducing capacity [14]. In this study, we measured the Fe^3+^-reducing activity of *P. ananatis* Sd-1 when it was cultured on different carbon sources. As shown in Fig. 3, although the Fe^3+^-reducing activities on rice straw and glucose substrates kept the same pattern of variation, the capacities were significantly increased (at least *P* < 0.01) in the presence of rice straw relative to glucose, especially on day 3. The significant induction of Fe^3+^-reducing activity throughout the biomass decomposition process supports our hypothesis that the Fenton chemistry system might play an important role in process of lignocellulose degradation by *P. ananatis* Sd-1. Besides, to confirm that if soluble iron ion was liberated from rice straw to cultures and was favorable for Fenton reaction, the total iron ion concentration in rice straw cultures was determined. As shown in Fig. 4, the total iron ion concentration kept increasing across the entire incubation when inoculated with *P. ananatis* Sd-1, while it was almost unchanging in control. The significant induction of Fe^3+^-reducing activity and increased total iron ion concentration throughout the biomass decomposition process supported our hypothesis that the Fenton chemistry system might play an important role in the process of rice straw degradation by *P. ananatis* Sd-1.

**Fig. 5:**
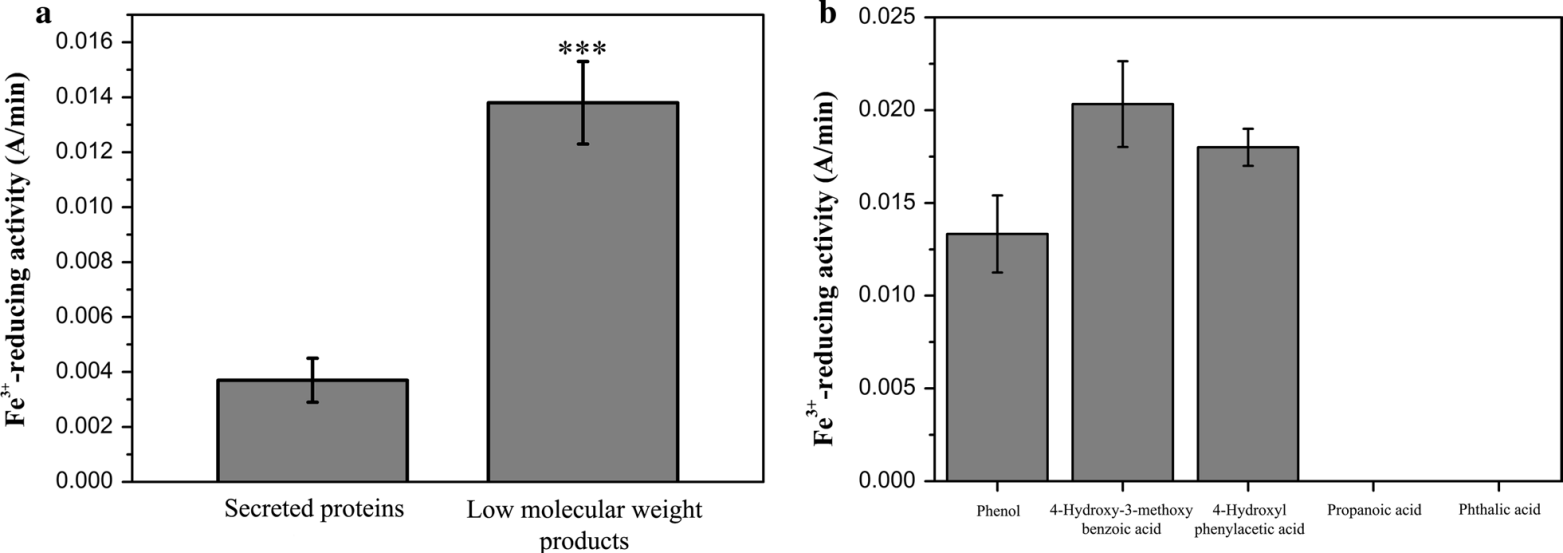
Fe^3+^-reducing activity of the secreted proteins and low molecular weight products extracted from *P. ananatis* Sd-1 cultures containing rice straw on day 3 (**a**). Fe^3+^-reducing activity of pure standards of phenol, 4-hydroxy-3-methoxybenzoic acid, 4-hydroxyphenylacetic acid, propanoic acid, and phthalic acid solutions (75 µM concentration) (**b**). The values represent the means of the three replicates with the standard deviation (SD). *Asterisks* represent significant differences from secreted proteins (statistical significance: ***P < 0.001)

In basidiomycete fungi, Fe^3+^ reduction is performed through three different mechanisms: (i) iron-reducing enzymes including ferric reductases and cellobiose dehydrogenase [37]; (ii) low molecular weight glycopeptides [38]; and (iii) redox cycling by low molecular weight metabolites, such as benzoquinones and involutin [35, 39, 40]. However, no genes encoding iron-reducing enzymes and low molecular weight glycopeptides were found in the genome of *P. ananatis* Sd-1 [17]. Therefore, we assume that Fe^3+^-reducing activity might be accomplished by extracellular low molecular weight products. Thus, both the secreted proteins and low molecular weight products from degraded rice straw were extracted from *P. ananatis* Sd-1 cultures on day 3 and were evaluated for Fe^3+^-reducing activity. As shown in Fig. 5a, the Fe^3+^-reducing activity value of low molecular weight products (0.0138 A/min) was significantly (*P* < 0.001) higher than that of the secreted proteins (0.0037 A/min).

Using qualitative GC–MS, we further identified the type of low molecular weight products from *P. ananatis* Sd-1-degraded rice straw. The total ion chromatograms (TIC) corresponding to extracted compounds from control and treated samples are shown in Additional file 6: Figure S5 and their peaks identity are listed in Table 1. A number of small molecular aromatic metabolites, such as phenol (RT 14.70), benzoic acid (RT 17.42), benzeneacetic acid (RT 17.79), acetophenone (RT 19.44), 4-hydroxy-3-methoxybenzoic acid (RT 20.42), phthalic acid (RT 20.91), 4-hydroxyphenylacetic acid (RT 21.58), and benzene (RT 22.25) which are considered as the basic units of the lignin polymer were only detected in the samples treated by *P. ananatis* Sd-1 for 3 days. In addition, some fatty acid compounds such as propanoic acid (RT 10.29), pentanoic acid (RT 12.54), octanoic acid (RT 17.37), decanoic acid (RT 18.13), nonanoic acid (RT 18.30), tetradecanoic acid (RT 21.45), and pentadecanoic acid (RT 21.94) which were generated through aromatic ring opening were also only presented in treated samples. The appearance of these metabolites confirmed again that the lignin structure of rice straw was decomposed by *P. ananatis* Sd-1. More importantly, we speculated that the Fe^3+^-reducing activity could be attributed to the presence of phenol, 4-hydroxy-3-methoxybenzoic acid, and 4-hydroxyphenylacetic acid which contained hydroxyl phenolic groups, due to the fact that hydroxybenzene derivatives have been found as the most efficient Fe^3+^-reductants [12, 41]. Aguiar et al. [42] showed that the identified 4-hydroxy-3-methoxybenzoic acid extracted from a wood chip culture treated with *Ceriporiopsis subvermispora* possesses Fe^3+^-reducing ability.

**Table 1:** Identification of metabolites as trimethylchlorosilane (TMS) derivatives from rice straw samples

Retention time	Compound	Control^a^	Treated^b^
10.29	Propanoic acid	**−**	**+**
12.54	Pentanoic acid	**−**	**+**
14.70	Phenol	**−**	**+**
17.37	Octanoic acid	**−**	**+**
17.42	Benzoic acid	**−**	**+**
17.79	Benzeneacetic acid	**−**	**+**
18.13	Decanoic acid	**−**	**+**
18.30	Nonanoic acid	**−**	**+**
19.44	Acetophenone	**−**	**+**
20.42	4-Hydroxy-3-methoxybenzoic acid	**−**	**+**
20.91	Phthalic acid	**−**	**+**
21.45	Tetradecanoic acid	**−**	**+**
21.58	4-Hydroxyphenylacetic acid	**−**	**+**
21.94	Pentadecanoic acid	**−**	**+**
22.25	Benzene	**−**	**+**
22.41	Hexadecanoic acid	**+**	**+**
23.51	Octadecanoic acid	**+**	**+**

^a^Non-inoculated (control) rice straw samples

^b^ *P. ananatis* Sd-1 degraded rice straw samples

Therefore, pure standards of phenol, 4-hydroxy-3-methoxybenzoic acid, and 4-hydroxyphenylacetic acid as well as another propanoic acid and phthalic acid (without hydroxyl phenolic groups) were assayed for Fe^3+^-reduction. As shown in Fig. 5b, phenol, 4-hydroxy-3-methoxybenzoic acid, and 4-hydroxyphenylacetic acid all exhibited distinct Fe^3+^-reduction capacities, while propanoic acid and phthalic acid could not be detected. The compound 4-hydroxy-3-methoxybenzoic acid showed the highest Fe^3+^-reducing ability among the three compounds, whereas lowest activity was detected for phenol. These results coincided with reports that compounds containing only one hydroxyl phenolic groups reacted weakly with iron compared to those containing two or three hydroxyl phenolic groups [12]. Our evidence suggests that hydroxyl phenolic groups containing aromatics in low molecular weight products are predominantly responsible for Fe^3+^-reducing activity. This provides evidence for the existence of Fenton-based reaction system in *P. ananatis* Sd-1 degradation process.

### Hydrogen peroxide production and consumption in *P. ananatis* Sd-1 degradation process

**Fig. 6:**
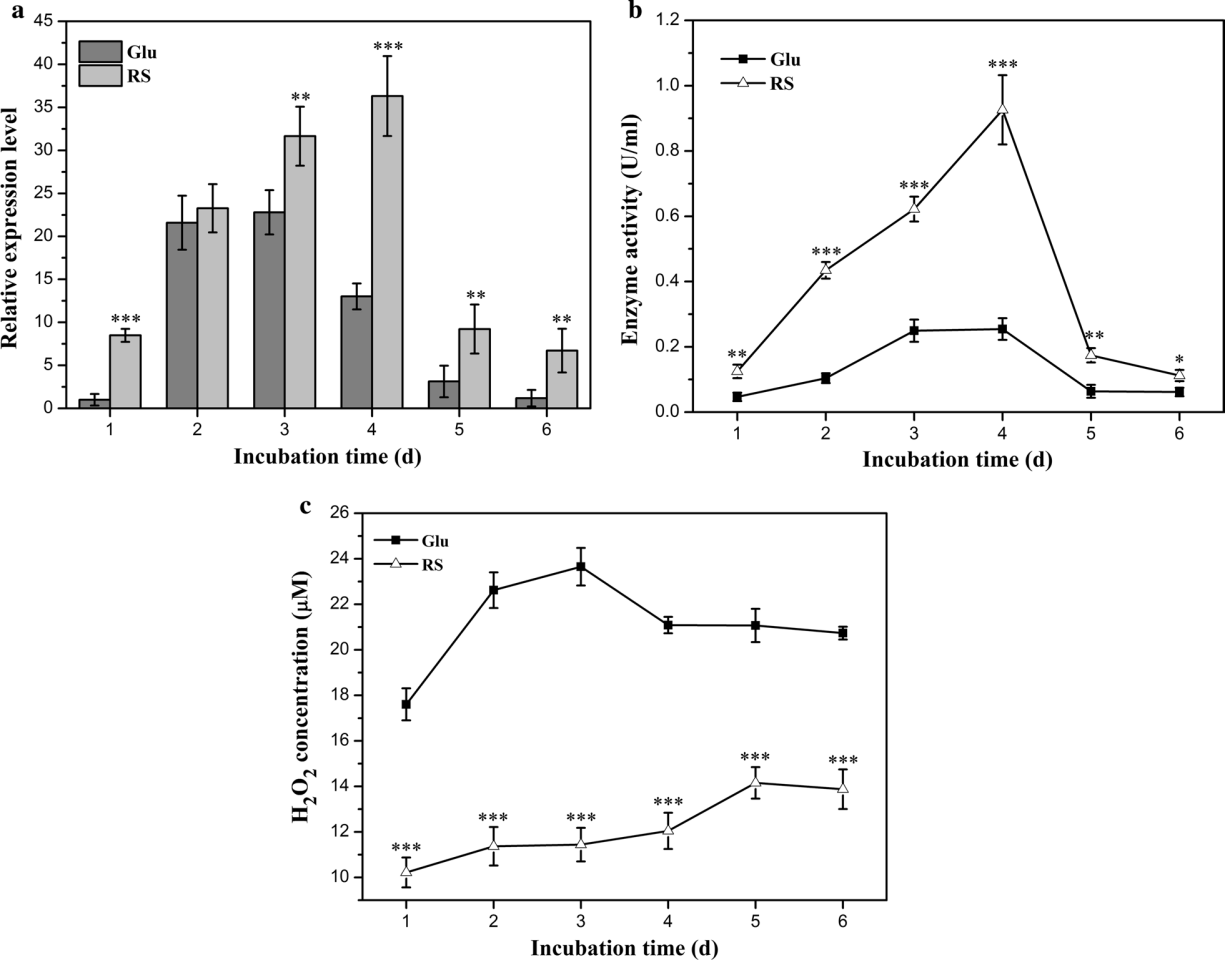
Hydrogen peroxide production and consumption analysis. GMC oxidoreductase gene (Y903_RS0107765) relative expression levels of *P. ananatis* Sd-1 grown in the presence of glucose (Glu) and rice straw (RS) substrates (**a**). Enzyme activity of GMC oxidoreductase during growth of *P. ananatis* Sd-1 on glucose (Glu) and rice straw (RS) substrates (**b**). Hydrogen peroxide concentration during growth of *P. ananatis* Sd-1 on glucose (Glu) and rice straw (RS) substrates (**c**). The values represent the means of the three replicates with the standard deviation (SD). *Asterisks* represent significant differences from the glucose cultures (statistical significance: *P < 0.05, **P < 0.01, ***P < 0.001)

In brown-rot fungi Fenton chemistry system, high levels of Fe^3+^-reducing activity and hydrogen peroxide generation lead to plant cell wall biodegradation. Hydrogen peroxide is metabolically generated by glucose-methanol-choline (GMC) oxidoreductases such as glucose oxidase, pyranose oxidase, and radical oxidases in basidiomycetes fungi [43]. In our previous bioinformatic analysis for *P. ananatis* Sd-1 genome, we identified a GMC oxidoreductase (GenBank: Y903_RS0107765) as the pyranose oxidase, which catalyzes the oxidation of d-glucose and several other aldopyranoses to yield 2-keto sugars and hydrogen peroxide [17, 44]. We monitored and compared the expression of this GMC oxidoreductase gene in cultures incubated with rice straw or glucose through quantitative real-time PCR (RT-qPCR). As shown in Fig. 6a, an increase in gene transcript levels was recorded for rice straw cultures until day 4. Across the entire incubation period with the exception of day 2, the gene transcript levels in rice straw cultures were significantly higher (at least *P* < 0.01) compared to glucose cultures. This suggested that the expression of *P. ananatis* Sd-1 GMC oxidoreductase gene was efficiently induced by lignocellulosic substrates. Activities of GMC oxidoreductase during growth of *P. ananatis* Sd-1 on glucose and rice straw substrates were then detected. As shown in Fig. 6b, the GMC oxidoreductase activities kept increasing until day 4 on both substrates, while it was significantly higher (at least *P* < 0.05) in rice straw cultures relative to glucose cultures during the whole incubation time. The variation tendency of GMC oxidoreductase activity was consistent with the gene expression levels results. We also assayed the concentration of hydrogen peroxide in rice straw and glucose cultures. As shown in Fig. 6c, the hydrogen peroxide concentration in rice straw cultures was significantly lower than that in glucose cultures (*P* < 0.001) throughout the entire incubation period. This is in contrast to the higher GMC oxidoreductase transcript levels and its enzyme activities in rice straw cultures. A similar observation was reported by Grinhut et al. [13] that hydrogen peroxide concentration in *Trametes* sp. M23 cultures was lower in the presence of humic acid degradation. Our observation indicates a linear relationship between increasing rice straw decomposition (Fig. 1b), Fe^3+^-reducing activities (Fig. 3), and the reduction of hydrogen peroxide during the incubation with *P. ananatis* Sd-1. It is suspected that lower hydrogen peroxide levels in rice straw cultures can be attributed to its growing consumption for reacting with Fe^2+^ to decompose lignocellulose. In addition, hydrogen peroxide is also an essential co-substrate for lignin peroxidase during lignin degradation, and so the loss of hydrogen peroxide might also be due to its role in enzymatic hydrolysis of lignin.

## Conclusion

The endophytic bacterium *P. ananatis* Sd-1 was shown to effectively degrade the natural lignocellulosic biomass, rice straw. The rate of rice straw degradation did not coincide with the lignocellulolytic enzymes activities and was significantly decreased in the presence of different hydroxyl radical scavengers. These alluded to the existence of a Fenton-based oxidation model, which was illustrated through the action of Fenton system-mediated hydroxyl radicals. The structural variation of rice straw constituents after treatment by *P. ananatis* Sd-1 was similar to that of Fenton reagent-treated samples. The crucial factors for Fenton chemistry, the Fe^3+^-reducing capacity, and hydrogen peroxide production (indicated by transcript levels of GMC oxidoreductase gene and its enzyme activities) in *P. ananatis* Sd-1 were significantly up-regulated in rice straw cultures. The low molecular weight products with hydroxyl phenolic groups might be responsible for Fe^3+^-reducing activity. The decrease of hydrogen peroxide in rice straw cultures relative to glucose cultures was consistent with the increasing rice straw degradation and Fe^3+^-reducing activities. These characteristics for Fenton reaction using Fe^2+^ and hydrogen peroxide confirmed the participation of Fenton chemistry system in the degradation process of rice straw by *P. ananatis* Sd-1. To our knowledge, this is the first discovery of a Fenton-based oxidation mechanism from a bacteria lignocellulose degradation system, which is valuable for the lignocellulosic bioenergy industry.

## Methods

### Growth of microorganism

*Pantoea ananatis* Sd-1 was obtained as previously reported [16]. Rice straw (obtained from Changsha, China) was washed thoroughly with distilled water, dried, and ground to pass through 40-mesh screen. *P. ananatis* Sd-1 was grown in the Luria–Bertani broth (LB) medium and incubated at 30 °C with shaking at 170 rpm to the logarithmic growth phase. Then 1 ml of culture was aseptically inoculated into 100 ml mineral salt medium containing rice straw (10.0 g rice straw, 2.0 g (NH_4_)_2_SO_4_, 1.0 g KH_2_PO_4_, 1.0 g KH_2_PO_4_, 0.2 g MgSO_4_, 0.1 g CaCl_2_, and 0.02 g MnSO_4_ in 1000 ml distilled water, pH 7.2), and incubated at 30 °C with shaking at 170 rpm for 6 days. Sterilized non-inoculated medium was used as control. All media were autoclaved at 121 °C for 20 min. Samples were withdrawn periodically at every 24 h and assessed for bacterial growth, reducing sugar concentration, enzymatic activity, and residual rice straw. All assays were performed with three replicates.

### Bacterial growth evaluation

The bacterial growth was assayed as previously described [17].

### Treatment of rice straw by Fenton reagent

One percentage rice straw (*w/v*) was added to Erlenmeyer flask (250 ml) with solutions containing 1.25 mM Fe^2+^ and 176 mM hydrogen peroxide, the pH of the reaction mixture was adjusted to 3.0 using oxalic acid and this mixture was stirred at 200 rpm for 2 h [45].

### The addition of hydroxyl radical scavenger in culture

Forty millimolar dimethyl sulfoxide (DMSO), mannitol, thiourea, and ethanol were added to the rice straw medium, respectively.

### Determination of residual rice straw

The residual rice straw biomass was harvested by vacuum filtration and washed twice with distilled water, then dried at 60 °C for 48 h. Cellulose, hemicellulose, and lignin contents of dried rice straw were determined by Van Soest method [46]. Cellulose content was calculated as the difference between acid detergent fiber (ADF) and acid detergent lignin (ADL). Hemicellulose content was calculated as the difference between neutral detergent fiber (NDF) and ADF, while lignin content was calculated as the difference between ADL and ash content. Rice straw biomass, cellulose, hemicellulose, and lignin-degrading efficiencies were calculated by the following formula:

$$\begin{aligned} &{\text{Rice straw}} \,\left( {{\text{cellulose}}, {\text{hemicellulose}}, {\text{lignin}}}\right) \; {\text{loss}} \;\left( \%\right) \\ & \qquad = \frac{{m_{0} - m_{n} }}{{m_{0} }} \times 100\;\% \end{aligned}$$

where *m*_0_ is the initial rice straw weight or (cellulose, hemicellulose, lignin) content, g; *m*_*n*_ is the nth day sampling rice straw weight or (cellulose, hemicellulose, lignin) in g.

### Reducing sugar and lignocellulolytic enzyme assay

The culture supernatant was collected by centrifugation at 12,000 rpm for 10 min and used for measurement of reducing sugar concentration, endoglucanase, exoglucanase, xylanase, lignin peroxidase, laccase, and Fe^3+^-reducing activity. Reducing sugar content was estimated by using DNS method with glucose as the standard [47]. Endoglucanase, exoglucanase, xylanase activities were determined by using carboxymethyl cellulose sodium salt (CMC-Na), filter paper (Whatman grade 1), and birchwood xylan as substrates, respectively [48, 49]. Concerning that a few of enzymes may be inevitably adsorbed on rice straw, the assayed enzymes in the cultures are proteins in systems except the adsorbed one by substrates. One unit of enzyme activity was defined as the amount of enzyme that catalyzes the release of 1 µM reducing sugar (glucose for endoglucanase and exoglucanase, xylose for xylanase) per minute under the specified assay conditions. Lignin peroxidase activity was assayed by monitoring oxidation of veratryl alcohol to veratraldehyde at 310 nm (ɛ310 = 9300 M^−1^ cm^−1^) according to the procedure of Chen et al. [22]. Laccase activity was performed according to the method of Nakagawa et al. [50] using 2,2,-azino-bis(3-ethylbenzothiazoline-6-sulfonic acid) (ABTS) as substrate and monitoring the production of ABTS radical at 420 nm (ɛ420 = 36,000 M^−1^ cm^−1^). One unit of lignin peroxidase or laccase activity was defined as the amount of enzyme required to produce 1 µM product per minute under the specified assay conditions.

### High-performance liquid chromatographic (HPLC) analysis of hydrolysate

A HPLC system (Agilent 1100) equipped with UV-detector for sugars (monitored at 245 nm), furfural, and 5-hydroxymethylfurfural (monitored at 230 nm) analysis. A ZORBAX Eclipse XDB-C18 column (150 mm × 4.6 mm × 5 µm) (Agilent, USA) was used for the analysis. Sugars were reacted with 1-phenyl-3-methyl-5-pyrazolone (PMP) for derivatization before detection. The derivatization procedure was performed as described by Dai et al. [51]. Sugars analysis was carried out with acetonitrile–water (80/20, *v*/*v*) as the mobile phase. The furfural and 5-hydroxymethylfurfural analysis were performed with acetonitrile–water (85/15, *v*/*v*) as the mobile phase. The flow rate of the eluent was 1 ml/min with a column temperature of 35 °C.

### Methane sulfinic acid assay

The produced methane sulfinic acid during the reaction of hydroxyl radical and DMSO was determined by addition of 1.0 ml sample and 2.0 ml 15 mM Fast Blue BB salt and reacted at room temperature in the dark, followed by extraction with 3.0 ml of toluene/butanol (3/1, *v*/*v*). The toluene/butanol phase was washed with 5 ml butanol-saturated water to remove the remaining unreacted diazonium salt and centrifuged. One milliliter of pyridine was added to upper phase to stabilize. The absorbance was determined at 420 nm against blanks containing all reagents except DMSO. Standards were prepared with 0–50 µM authentic methane sulfinic acid [52].

### FTIR spectroscopy analysis

Samples for FTIR analysis were vacuum-dried and milled into a powder, then mixed with dry KBr at a ratio of 1/200 (*w*/*w*) and homogenized into a very fine powder. FTIR spectroscopy analysis was performed using a Varian 2000 FTIR spectrometer, taking 32 scans for each sample at 4 cm^−1^ resolution, ranging from 400 to 4000 cm^−1^.

### Py-GC/MS analysis

Py-GC/MS analysis was performed using a EGA/PY-3030D pyrolyzer (Frontier Laboratories, Japan) coupled to a QP2010 ultra gas chromatograph-mass spectrometer (Shimadzu, Japan) with a DB-5 capillary column (30 m × 0.25 mm × 0.25 µm) (Agilent, Palo Alto, USA). The pyrolysis was initially set at 200 °C and held for 1 min, then increased to 600 °C at 20 °C/min and held for 12 s. The GC temperature was started at 80 °C and held for 1 min, later on increased to 280 °C at 5 °C/min and held for 10 min. The mass spectra of each compound were identified according to the National Institute of Standards and Technology (NIST) library. Quantification of pyrolysis products was based on the peak area of characteristic fragment ions. The relative abundances of each pyrolysis products were calculated as the percentage of the sum of all peak areas.

### GC–MS analysis degradation products

Cultures of *P. ananatis* Sd-1 grew in rice straw were collected on day 3, then centrifuged at 8000 rpm for 15 min to remove biomass. Thereafter, supernatants were treated according to Raj et al. [53]. Briefly, supernatants were acidified to pH 1–2 with 12 M HCl and then extracted with equal volumes of ethyl acetate three times. Extracted liquor was collected and dewatered over anhydrous Na_2_SO_4_. The extract liquor was filtered through filter paper and concentrated to approximately 1 ml by rotary vacuum evaporator. Then 100 µl dioxane and 10 µl pyridine were added to the sample followed by silylation with 50 µl of trimethyl silyl [(*N*,*O*-bis(trimethylsilyl) trifluoroacetamide) BSTFA/trimethylchlorosilane (TMS) = 99/1 (*v*/*v*)]. The mixture was heated at 60 °C for 15 min with periodic shaking to dissolve residue. GC–MS analysis of the silylated compounds was performed according to the procedure of Chen et al. [22]. The identification of TMS derivatives derived from bacterial degradation was conducted by comparing their mass spectra with that of the NIST library available on the instrument and by comparing the retention time to the original standards.

### Determination of Fe^3+^-reducing activity

The Fe^3+^-reducing activity was performed by monitoring Fe^3+^-reduction based on formation of the ferrozine-Fe^2+^ complex [10]. The reaction mixture (3 ml) contained 50 µM FeCl_3_, 10 mM ferrozine in 1 M acetic acid, 1 M sodium acetate buffer (pH 4.5), and 2 ml sample. The change in absorbance of reaction at 562 nm was immediately recorded using a UV-visible spectrophotometer within 3 min. One unit of Fe^3+^-reducing activity was defined as the rate of absorbance increase at 562 nm per minute.

### Total iron ion concentration assay

Total iron ion concentration was determined by a colorimetric ferrozine assay [54]. One hundred milliliters of the sample were mixed with 100 µl 10 mM HCl. Then 100 µl of the iron-detection reagent (6.5 mM ferrozine, 6.5 mM neocuproine, 2.5 M ammonium acetate, and 1 M ascorbic acid dissolved in water) was added to the mixture. After 30 min incubation, absorbance was immediately recorded at 562 nm using a UV-visible spectrophotometer. Standard FeCl_3_ (0-50 µM) was used to quantify sample measurements.

### Total secreted proteins extraction

Cultures of *P. ananatis* Sd-1 grew in rice straw were collected on day 3. After filtering, biomass and supernatant were separated by centrifugation (4000 rpm, 10 min, 4 °C) and the supernatant was further clarified by filtration through 0.2 µm membranes (Millipore corporation, Billerica, MA). The supernatant protein fraction was concentrated using acetone precipitation [55]. The precipitated protein pellets were dissolved in an appropriate volume of phosphate buffer solution (0.02 M, pH 7.2), then used for the Fe^3+^-reducing activity assays. Protein concentrations were determined using BCA protein assay kit (Dingguo, Beijing, China).

### Total RNA extraction and complementary DNA synthesis

The bacterial cells were harvested from rice straw- and glucose-containing cultures at every 24 h intervals by centrifugation at 4000 rpm for 10 min at 4 °C. Total RNA was extracted with TRIzol reagent (Invitrogen, Carlsbad, USA) according to the manufacturer’s specifications. Amplification of complementary DNA (cDNA) was carried out using PrimeScript RT reagent Kit (Takara, Dalian, China) according to the manufacturer’s instructions.

### Quantitative real-time PCR

Primers used for quantitative real-time PCR (RT-qPCR) were designed using Beacon Designer 7.7 software (Additional file 7: Table S2). The RT-qPCR reactions were carried out using Mx3000P thermal cycler (Stratagene, Santa Clara, CA) and normalized using 16S ribosomal RNA expression levels as the internal reference. Reactions were conducted using SYBR^®^ Premix Ex Taq ™ II (Tli RnaseH Plus) (Takara, Dalian, China) with 20 µl reaction mixture containing 10 µl of 2 × SYBR Premix Ex Taq II, 0.4 µM of primers, 0.05 µg of cDNA template, and nuclease-free water. The applied cycling conditions were as follows: initial denaturation at 95 °C for 30 s, followed by 40 cycles of 95 °C for 5 s, then 60 °C for 30 s, and 72 °C for 30 s. A melting curve analysis was performed after amplification reaction to verify primer specificity. The specificity of the primers was confirmed by the presence of a single peak of the melting curve. All reactions were performed in triplicate. Relative transcript levels were determined by 2^−ΔΔCt^ method as described previously: normalizing the gene expression levels in rice straw-containing culture to that in glucose-containing culture, where expression level of the gene in glucose-containing culture at the first day was set to one [17].

### Glucose-methanol-choline (GMC) oxidoreductase activity assay

The intracellular proteins from *P. ananatis* Sd-1 were extracted from harvested bacterial cells from rice straw- and glucose-containing cultures at every 24 h intervals by sonication, and centrifuged (12,000 rpm, 4 °C, 10 min). GMC oxidoreductase activity was determined like pyranose oxidase by monitoring the production of ABTS radical at 420 nm (ɛ420 = 36,000 M^−1^ cm^−1^) based on a coupled reaction of horseradish peroxidase and its substrate ABTS as Leitner et al. [56] described. One unit of enzyme activity was defined as the amount of enzyme required for oxidation of 2 µM ABTS per minute under the assay conditions described above.

### Hydrogen peroxide concentration determination

Cultures of *P. ananatis* Sd-1 that grew in rice straw and glucose media were harvested at 24 h intervals, centrifuged (8000 rpm, 4 °C, 5 min), and supernatants collected. A hydrogen peroxide assay kit (Beyotime Institute of Biotechnology, Haimen, China) was used for determination of hydrogen peroxide concentration according to the manufacturer’s recommended protocol.

## Additional files

10.1186/s13068-016-0623-x Growth of bacterial strain and reducing sugars production during the degradation of rice straw by *P. ananatis* Sd-1 in the presence of DMSO.

10.1186/s13068-016-0623-x The percentage of weight loss for total rice straw, cellulose, hemicellulose and lignin in the presence of mannitol (RS + mannitol), thiourea (RS + thiourea), ethanol (RS + ethanol) and absence (RS) of hydroxyl radical scavenger after 6 days incubation (a); enzymes activities of endoglucanase, exoglucanase, xylanase, lignin peroxidase and laccase during the degradation of rice straw by *P. ananatis* Sd-1 in the presence of mannitol (b), thiourea (c) and ethanol (d).

10.1186/s13068-016-0623-x Sugars, furfural and 5-hydroxymethylfurfural (HMF) derived from rice straw during the *P. ananatis* Sd-1 and Fenton reagent treatment.

10.1186/s13068-016-0623-x Determination of methane sulfinic acid in the presence of DMSO during the degradation process of rice straw by *P. ananatis* Sd-1.

10.1186/s13068-016-0623-x FTIR spectra of rice straw before (control) and after 6 days treatment by *P. ananatis* Sd-1 or Fenton reagent. The black arrow indicated the appearance of new peak located in the carbonyl groups region.

10.1186/s13068-016-0623-x Total ion chromatograph (TIC) of ethyl acetate extract analysed as TMS derivatives from control (a) and *P. ananatis* Sd-1 degraded rice straw after 3 days incubation (b).

10.1186/s13068-016-0623-x Primers used for quantitative real time-PCR.

## Abbreviations

Py-GC/MS: pyrolysis gas chromatography–mass spectrometry
FTIR: Fourier transform infrared spectroscopy
GMC: glucose-methanol-choline
DMSO: dimethylsulfoxide
GC–MS: gas chromatography–mass spectrometry
TIC: total ion chromatogram
RT: retention time
RT-qPCR: quantitative real-time polymerase chain reaction
ADF: acid detergent fiber
ADL: acid detergent lignin
NDF: neutral detergent fiber
HPLC: high-performance liquid chromatographic
PMP: 1-phenyl-3-methyl-5-pyrazolone
