# Supplementary figures and images for: Involvement of Fenton chemistry in rice straw degradation by the lignocellulolytic bacterium Pantoea ananatis Sd-1
Source: Biotechnol Biofuels. 2016 Oct 6;9:211. doi: 10.1186/s13068-016-0623-x (PMC5054592; doi:10.1186/s13068-016-0623-x)

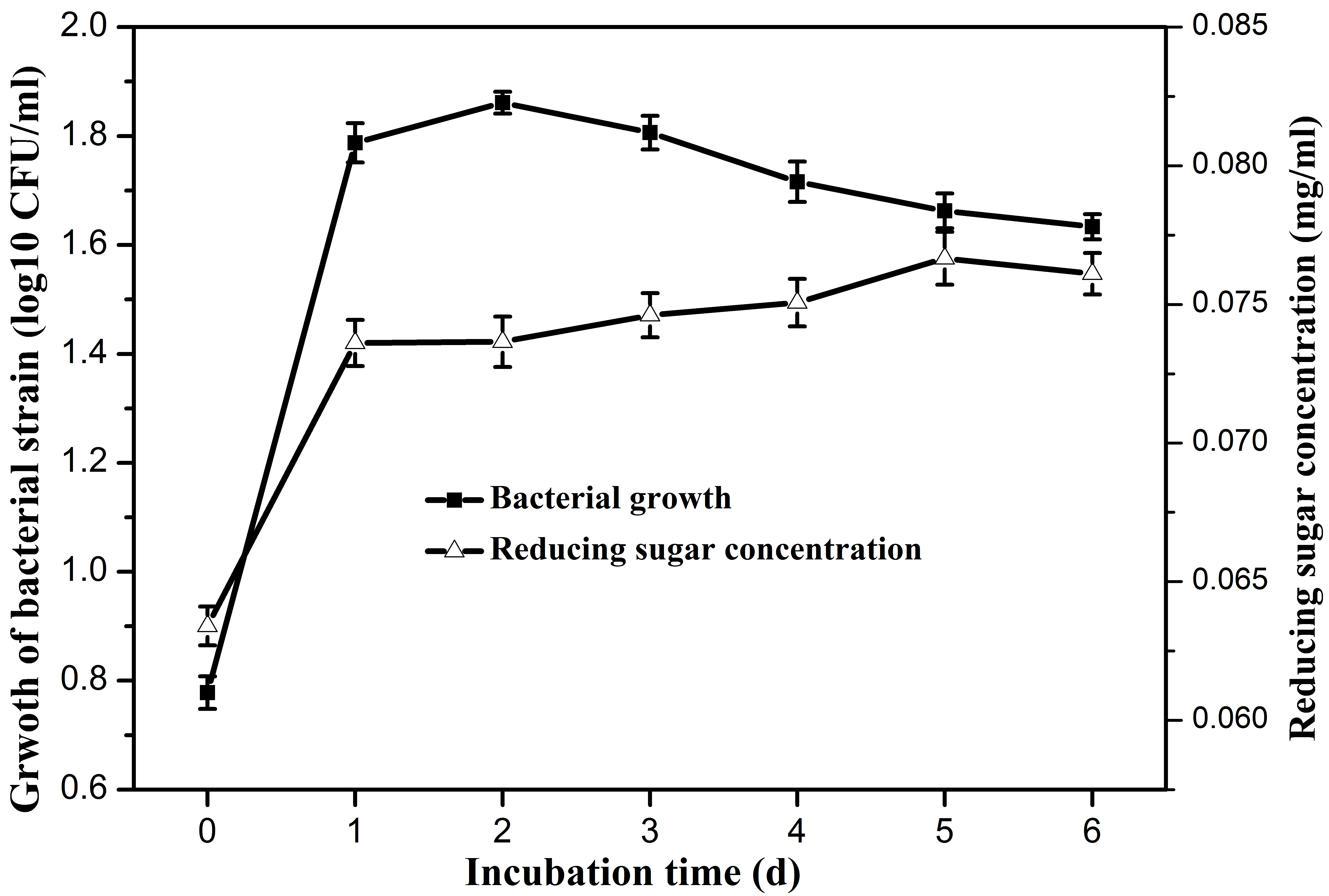

Supplement: Supplementary file 1 — 10.1186/s13068-016-0623-x Growth of bacterial strain and reducing sugars production during the degradation of rice straw by P. ananatis Sd-1 in the presence of DMSO. [file 13068_2016_623_MOESM1_ESM.tif]

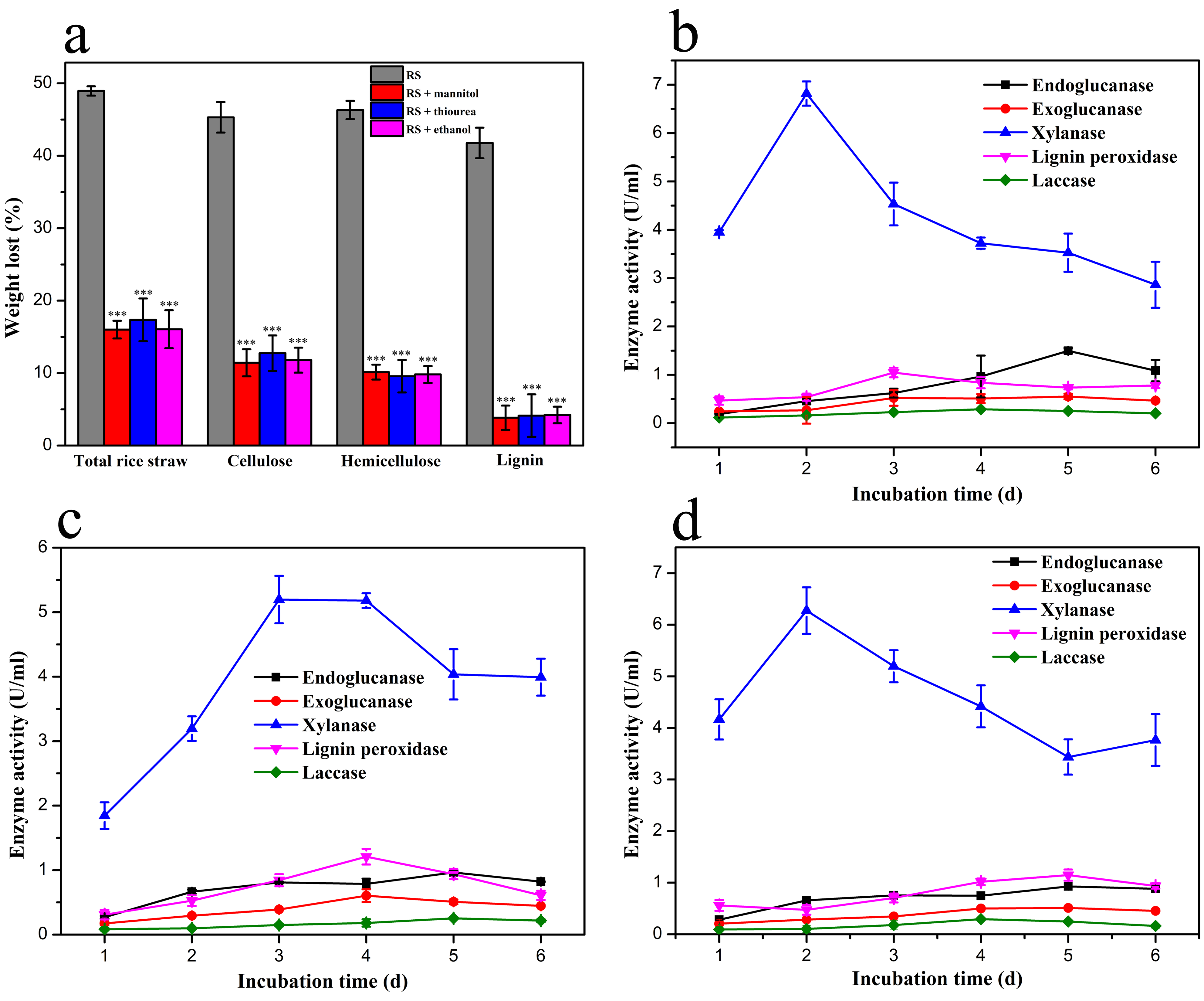

Supplement: Supplementary file 2 — 10.1186/s13068-016-0623-x The percentage of weight loss for total rice straw, cellulose, hemicellulose and lignin in the presence of mannitol (RS + mannitol), thiourea (RS + thiourea), ethanol (RS + ethanol) and absence (RS) of hydroxyl radical scavenger after 6 days incubation (a); enzymes activities of endoglucanase, exoglucanase, xylanase, lignin peroxidase and laccase during the degradation of rice straw by P. ananatis Sd-1 in the presence of mannitol (b), thiourea (c) and ethanol (d). [file 13068_2016_623_MOESM2_ESM.tif]

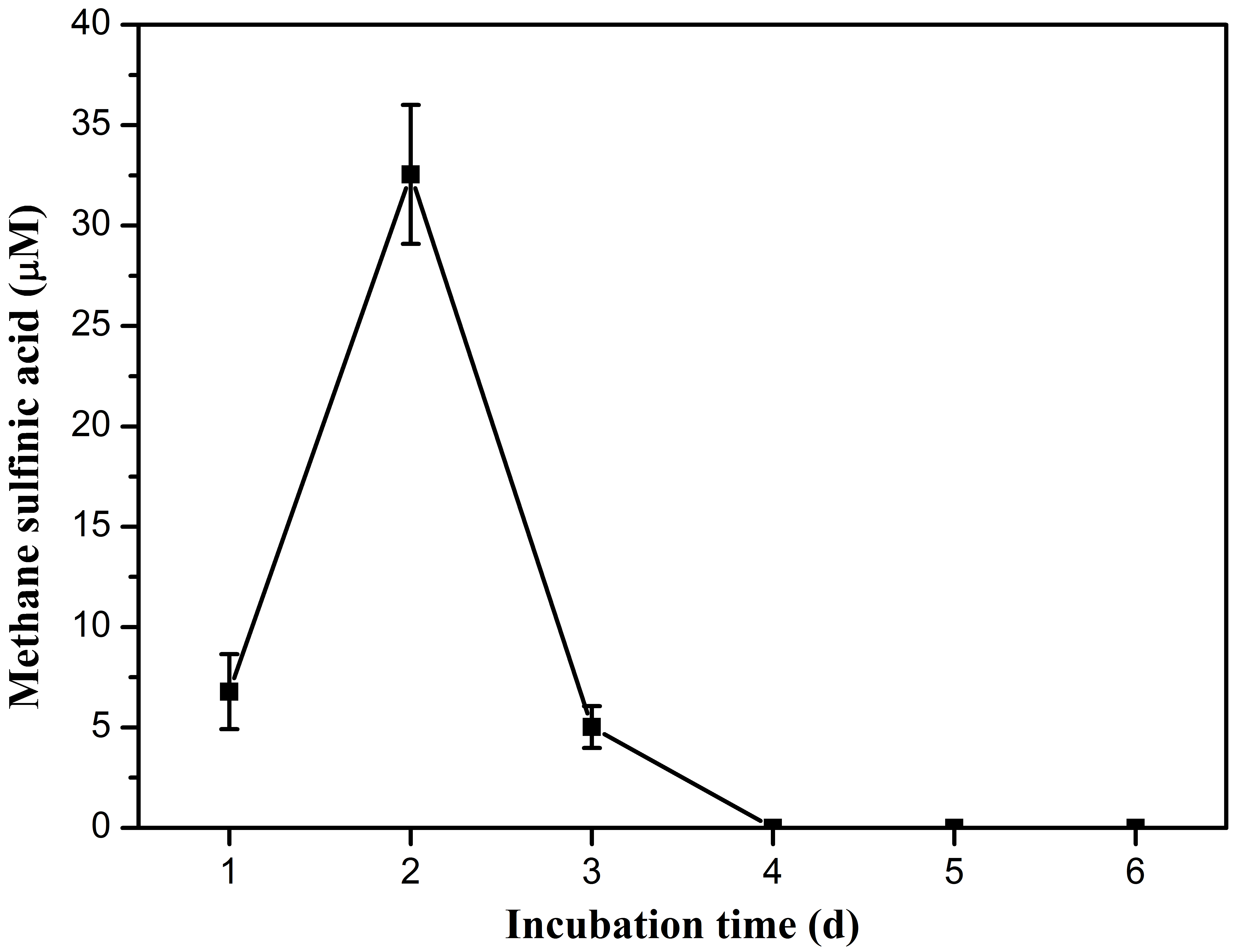

Supplement: Supplementary file 4 — 10.1186/s13068-016-0623-x Determination of methane sulfinic acid in the presence of DMSO during the degradation process of rice straw by P. ananatis Sd-1. [file 13068_2016_623_MOESM4_ESM.tif]

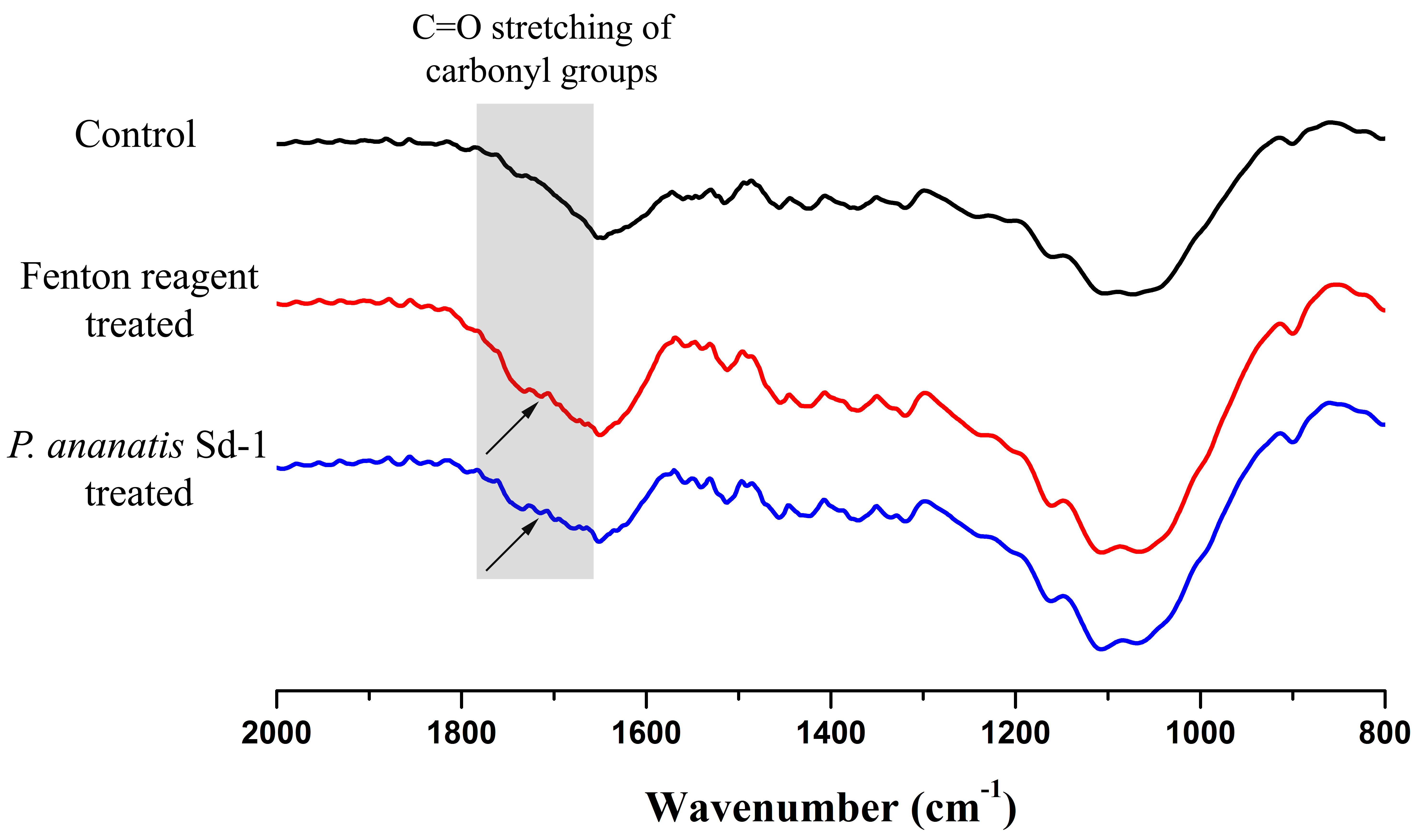

Supplement: Supplementary file 5 — 10.1186/s13068-016-0623-x FTIR spectra of rice straw before (control) and after 6 days treatment by P. ananatis Sd-1 or Fenton reagent. The black arrow indicated the appearance of new peak located in the carbonyl groups region. [file 13068_2016_623_MOESM5_ESM.tif]

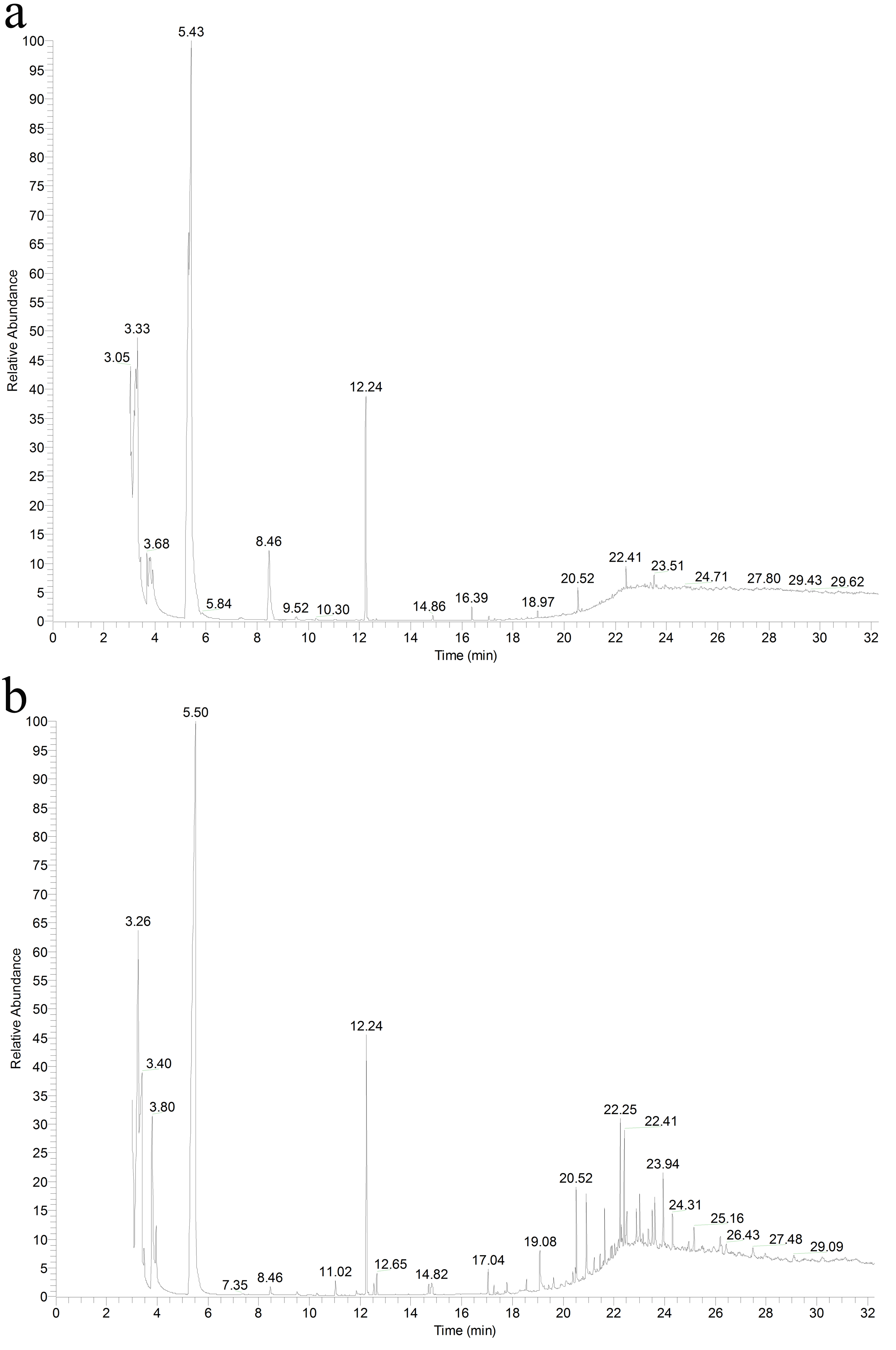

Supplement: Supplementary file 6 — 10.1186/s13068-016-0623-x Total ion chromatograph (TIC) of ethyl acetate extract analysed as TMS derivatives from control (a) and P. ananatis Sd-1 degraded rice straw after 3 days incubation (b). [file 13068_2016_623_MOESM6_ESM.tif]
