# Supplementary material for: Involvement of Fenton chemistry in rice straw degradation by the lignocellulolytic bacterium Pantoea ananatis Sd-1
Source: Biotechnol Biofuels. 2016 Oct 6;9:211. doi: 10.1186/s13068-016-0623-x (PMC5054592; doi:10.1186/s13068-016-0623-x)
Supplement: Supplementary file 3 — 10.1186/s13068-016-0623-x Sugars, furfural and 5-hydroxymethylfurfural (HMF) derived from rice straw during the P. ananatis Sd-1 and Fenton reagent treatment. [file 13068_2016_623_MOESM3_ESM.pdf]

Table S1 Sugars, furfural and 5-hydroxymethylfurfural (HMF) derived from rice straw during the *P. ananatis* Sd-1 and Fenton reagent treatment.

	Time	Glucose (g/l)	Xylose (g/l)	Arabinose (g/l)	Furfural (g/l)	HMF (g/l)
**RS**	**1 d**	0.0245	0.0272	0.0153	0.037	ND[a]
	**2 d**	0.0421	0.0187	0.0386	0.018	ND
	**3 d**	0.0353	0.0318	0.0175	ND	ND
	**4 d**	0.0482	0.0230	0.0336	ND	ND
	**5 d**	0.0360	0.0194	0.0227	ND	ND
	**6 d**	0.0251	0.0283	0.0346	ND	ND
**RS + DMSO**	**1 d**	0.0147	0.0182	0.0232	ND	ND
	**2 d**	0.0129	0.0256	0.0317	ND	ND
	**3 d**	0.0244	0.0187	0.0239	ND	ND
	**4 d**	0.0355	0.0221	0.0141	ND	ND
	**5 d**	0.0192	0.0205	0.0201	ND	ND
	**6 d**	0.0186	0.0237	0.0262	ND	ND
**Control[b]**		0.0115	ND	ND	ND	ND
**Fenton reagent**	**2 h**	0.0354	0.0266	0.0223	0.195	0.137

[a] Not detected

[b] Non-inoculated cultures
