# Supplementary material for: Involvement of Fenton chemistry in rice straw degradation by the lignocellulolytic bacterium Pantoea ananatis Sd-1
Source: Biotechnol Biofuels. 2016 Oct 6;9:211. doi: 10.1186/s13068-016-0623-x (PMC5054592; doi:10.1186/s13068-016-0623-x)
Supplement: Supplementary file 7 — 10.1186/s13068-016-0623-x Primers used for quantitative real time-PCR. [file 13068_2016_623_MOESM7_ESM.pdf]

**Table S2** Primers used for quantitative real time-PCR

| **Gene (CDS no.)** | **Sequence of 5'** | **Sequence of 3'** | **Production length** |
|---|---|---|---|
| GMC family oxidoreductase gene (GenBank: Y903_RS0107765) | TTCCAGGACC TGTCTAAG | TACGCAGTTT CAGCTCTA | 169 bp |
| 16S rRNA gene (GenBank: Y903_RS0109280) | AAGTCGGAAT CGCTAGTA | TACGGTTACC TTGTTACG | 182 bp |
